# Primary Intracranial Synovial Sarcoma

**DOI:** 10.1155/2016/5608315

**Published:** 2016-05-09

**Authors:** Mohit Patel, Luyuan Li, Ha Son Nguyen, Ninh Doan, Grant Sinson, Wade Mueller

**Affiliations:** Department of Neurosurgery, Medical College of Wisconsin, Milwaukee, WI 53226, USA

## Abstract

*Background*. Synovial sarcoma is an aggressive soft tissue sarcoma with uncertain histological origin. The pathology frequently presents as a localized disease, especially near large joints around the knee and thigh. Intracranial disease, which is rare, has been reported as metastasis from synovial sarcoma. We report a case with no obvious primary extracranial pathology, suggesting primary intracranial disease; this has not been reported in the literature.* Case Description*. A 21-year-old male, with a prior right skull lesion resection for atypical spindle cell neoplasm, presented with headaches, gait instability, left arm weakness, and left homonymous hemianopsia. CT of head demonstrated a right parietal hemorrhagic lesion with mass effect, requiring surgical decompression. Histopathology revealed synovial sarcoma. FISH analysis noted the existence of the t(X;18)(p11.2;q11.2) chromosomal translocation. PET scan did not show other metastatic disease. He underwent stereotactic radiotherapy and adjuvant chemotherapy. At 2-year follow-up, he remained nonfocal without recurrence.* Conclusion*. We report the first known case of primary intracranial synovial sarcoma. Moreover, we stress that intracranial lesions may have a tendency for hemorrhage, requiring urgent lifesaving decompression.

## 1. Background

Synovial sarcoma is an aggressive soft tissue sarcoma with uncertain histological origin. Its trademark is a unique t(X;18)(p11.2;q11.2) chromosomal translocation resulting in SYT-SSX fusion protein. The pathology frequently presents as a localized disease, especially near large joints around the knee and thigh [1]. Intracranial disease, which is rare, has been reported as metastasis from synovial sarcoma [1–8]. We report a case with no obvious primary extracranial pathology, suggesting primary intracranial disease; this has not been reported in the literature.

## 2. Case Presentation

**Figure 1:**
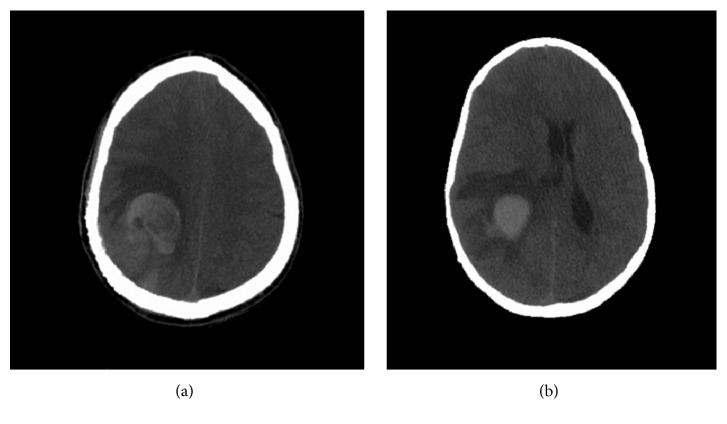
Axial CT of head for patient demonstrates right parietal heterogeneous, hyperdense mass with a large medial hematoma.

A 21-year-old male presented with persistent headaches. CT of head showed a right parietal lobulated skull lesion with intracranial extensions. The lesion was subsequently resected and diagnosed as atypical spindle cell neoplasm. Eight months later, patient presented to the emergency room with headaches, gait instability, and left arm weakness. Physical examination revealed left homonymous hemianopsia, left hand weakness, and ataxia. Patient could not tolerate MRI due to agitation. CT of head demonstrated a right parietal heterogeneous, hyperdense mass with a large medial hematoma. Due to increased agitation, repeat CT of head was completed 8 hours later, which showed worsening midline shift to 9 mm (Figure 1). Patient was taken to the operating room emergently for decompression and clot evacuation. The mass was friable and hemorrhagic. Postoperatively, patient's visual field gradually improved and left hand weakness resolved. He was discharged home 3 days later. Histopathology was consistent with synovial sarcoma. FISH analysis noted the existence of the t(X;18)(p11.2;q11.2) chromosomal translocation. PET scan did not show any other metastatic disease.

One month after discharge, the patient underwent stereotactic radiotherapy (60 Gy in 30 fractions) for local tumor control. Three weeks after completion of radiotherapy, he had another operation for excision of the residual tumor and cranioplasty. Intraoperatively, multiple lobulated cysts were seen and removed. Gross total resection of the tumor was achieved; a wire mesh was placed over the right parietal bony defect. The patient recovered quickly and went home on postoperative day 3. Two months later, the patient received 3 cycles of adjuvant chemotherapy (AIM regimen) consisting of doxorubicin (adriamycin), ifosfamide, and mesna. Since then, the patient has been followed up closely in the clinic with MRI scans every 3 months. He continued to be neurologically intact without any evidence of tumor recurrence two years after the chemotherapy.

## 3. Discussion

Sarcoma is generally categorized into bone and soft tissue sarcoma. Synovial sarcoma is a type of soft tissue sarcoma that occurs mainly in adolescents and young adults between the ages of 15 and 30 years, with a slight male predominance [1, 10]. The neoplasm constitutes 5 to 10% of the soft tissue sarcomas [1, 2]. There are four subtypes of synovial sarcoma: monophasic, monophasic epithelial, biphasic, and poorly differentiated [1, 11]. The term “synovial sarcoma” was introduced in 1934 due to similarities with synovial tissue under light microscopy [1]. However, subsequent immunohistochemical and ultrastructural studies demonstrated that tumor cells do not share characteristics with normal synovium [12]. Moreover, cDNA-microarray based studies suggest a close linkage between synovial sarcoma and neural crest-derived malignant peripheral nerve sheath tumor [10, 13]. Other studies also indicate that a human multipotent mesenchymal stem cell can function as a cell of origin [14].

Similar to other soft tissue sarcomas, the most common initial symptom of synovial sarcoma is an enlarging soft tissue mass [4]. Frequently seen near large joints, synovial sarcoma may also arise primarily in a wide variety of organs, including kidney [15], heart [16], and lung [1]. A small minority of patients develop symptoms secondary to metastatic lesions prior to diagnosis of the primary pathology, largely complaints related to lung metastases [4, 10]. The rate for metastatic disease in synovial sarcoma ranges up to 33% [4, 17]. Common sites of metastasis include lung, bone, and lymph nodes [2, 10]. Intracranial disease, which is rare, has been reported as metastasis from synovial sarcoma. These are summarized in Table 1. Of the available data on six cases with intracranial metastases, one exhibited a left soft tissue mass without neurologic symptoms [2], one demonstrated sensory aphasia [9], and two patients exhibited headaches/vomitus [4, 5]; for the remaining two cases, the symptoms were not clear based on review of the pertinent articles [1, 3]. Our patient presented with a right skull lesion with intracranial extension and headaches; his recurrence was associated with neurological deficits and a hemorrhagic tumor with significant mass effect. Along with the case by Przkora et al. [4], this is the second instance that documents intracranial hemorrhage associated with synovial sarcoma. Unlike prior cases, this case highlights intracranial disease without obvious primary extracranial pathology, suggesting primary intracranial disease.

The standard treatment for local synovial sarcoma is surgical resection with wide margins [1]. Adjuvant therapies, including radiation and chemotherapy, have demonstrated benefits for local recurrence and prognosis [3]. Treatment for metastatic intracranial disease has not been optimized. Siegel et al. [2] reported a patient with a skull lesion that appeared to respond to neoadjuvant chemotherapy and external beam radiation; there was no evidence of recurrence in the cranial region at the time of the patient's death (he succumbed to pulmonary and intra-abdominal metastases). Kaufman and Tsukada [5] reported a patient with cerebral metastasis to the right cerebellum who was treated with radiation and documented complete remission; the patient passed away from pulmonary metastases; however, at the time of autopsy residual tumor was found in the brain [5]. Nuwal et al. [1] mentioned that their patient had chemotherapy with temozolamide and radiation to the skull; the effectiveness of the treatment was unclear, as the patient succumbed 6 months later due to pleural effusion/ascites/anasarca. Flannery et al. [6] described a patient who passed away 1 month after her diagnosis of brain metastases after receiving surgical resection followed by gamma knife. Grossman and Ram [7] reviewed 21 patients with intracranial metastases from sarcoma, including 4 with synovial sarcoma; their patients received surgical resection, whole brain radiation, and/or stereotactic radiosurgery; median overall survival was 7 months. Three additional cases did not comment on adjuvant therapy after surgical treatment [3, 4, 8] and one [9] was not available in English. Given the paucity of clinical data available regarding this deadly disease, it is vital to collect and report as much clinical information as possible so this disease can be further dissected, which ultimately will help to devise new therapies.

## 4. Conclusion

Synovial sarcoma is a malignant neoplasm. Rare instances of intracranial disease have been attributed to metastasis. Our patient is the first known case of intracranial disease without obvious primary extracranial pathology, suggesting that the primary disease arose from the brain. We also noted the hemorrhagic complication associated with brain lesions. In the case of sudden neurological deterioration, it is important to be cognizant of the tendency for intracranial lesions to hemorrhage, requiring urgent lifesaving decompression.

## Figures and Tables

**Table 1:** Literature review. Grossman and Ram [7], Yoshida et al. [8], and Baptista et al. [3] mentioned 5 more patients collectively without clinical detail. *∗∗∗* No available data.

Literature	Year	Age	Gender	Primary	Symptoms at presentation	Intracranial findings	Other sites of metastases	Outcome
Flannery et al. [6]	2010	26	F	Knee	Hemiparesis	*∗∗∗*	*∗∗∗*	Survival 1 month
Kaufman and Tsukada [5]	1976	32	M	Right foot	Headaches, vomiting, ataxia	Right cerebellar mass	Lung, “numerous subcutaneous masses”	Survival 7 months
Nuwal et al. [1]	2012	35	M	Left lung	*∗∗∗*	Left parietooccipital mass	None	Survival 6 months
Otani et al. [9]	2013	41	F	Left inguinal region	Sensory aphasia	Left frontal, parietal, parietotemporal	*∗∗∗*	*∗∗∗*
Przkora et al. [4]	2003	74	F	Right popliteal mass	Headaches, vomiting	right frontal mass with hemorrhage	Lung	Survival 1 year
Siegel et al. [2]	2008	17	M	Right thigh	Left soft tissue mass, otherwise no neurologic symptoms	Left skull mass	Femur, buttock, intra-abdominal, lung	Survival 2 years
Our case	2015	21	M	Right skull lesion with intracranial extension	Headaches, ataxia, left hemianopsia left arm weakness	Right parietal mass with hemorrhage	None	At least 2 years
